# Resolution of biliary stricture after living donor liver transplantation in a child by percutaneous trans-hepatic cholangiography and drainage: a case report

**DOI:** 10.1186/1752-1947-7-160

**Published:** 2013-06-20

**Authors:** Gabriel Putzer, Peter Paal, Andreas P Chemelli, Walter Mark, Wolfgang Lederer, Franz J Wiedermann

**Affiliations:** 1 Department of Anaesthesiology and Critical Care Medicine, Innsbruck Medical University, Anichstrasse 35, 6020, Innsbruck, Austria; 2 Department of Radiology, Innsbruck Medical University, Anichstrasse 35, 6020, Innsbruck, Austria; 3 Department of Abdominal, Thoracic and Transplant Surgery, Innsbruck Medical University, Anichstrasse 35, 6020, Innsbruck, Austria

## Abstract

**Introduction:**

Intra-hepatic cholestasis arising from biliary strictures is a frequent complication in pediatric patients after liver transplantation. Minimally invasive procedures such as percutaneous drainage placement and balloon dilation are the preferred diagnostic and therapeutic modalities.

**Case presentation:**

We report the case of a 12-month-old Caucasian boy with biliary atresia who was initially treated with hepatoportoenterostomy. In the following months, he developed biliary cirrhosis, accompanied by cystic bile retention, recurrent bile duct infections and malabsorption. Six months after the initial surgical intervention, he underwent living donor liver transplantation. Within two months, the hepatico-jejunostomy became occluded leading to progressive intra-hepatic cholestasis. Under sonographic guidance, external drainage of bile was accomplished by percutaneous trans-hepatic cholangiography and drainage. In total, our patient underwent 12 interventions under general anesthesia until balloon dilatation of the hepatico-jejunostomy was successfully performed. Finally, our patient’s general condition improved and he gained weight.

**Conclusions:**

Minimally invasive techniques are preferred to surgical revisions and justify even multiple attempts. Interventions under general anesthesia, though not without risks, are still reasonable. Co-operation with parents and multidisciplinary approach to complication management by the involved surgeon, radiologist, pediatrician and anesthesiologist are important.

## Introduction

Liver transplantation has been successfully performed in pediatric patients with end-stage liver disease. A significant advance in transplantation has been the use of living donor and split-liver grafts. Technical challenges include vascular anatomy, sufficient volume for the metabolic demands of the patient, and biliary drainage [1]. Transplantation requires vascular reunion of the portal vein, hepatic artery and hepatic veins between graft and host. In addition, continuity of the bile duct with the gastro-intestinal tract has to be established, for example, via hepatico-jejunostomy.

Post-transplantation biliary strictures occur in up to one-third of pediatric patients who undergo liver transplant, and cases are conventionally managed by interventional radiological techniques [2].

## Case presentation

We report the case of a 12-month-old Caucasian boy, who was treated at the Department of Interventional Radiology for biliary stricture following living donor liver transplantation (LDLT). During the post-partum period, our patient developed fever and presented with increased liver enzymes and progressive conjugated hyperbilirubinemia. Ultrasound examination of the abdomen revealed complete obliteration of the extra-hepatic biliary system indicating type III biliary atresia.

At an age of four months, hepatoportoenterostomy (Kasai procedure) was performed to allow for bile drainage [3]. A histopathological examination revealed fibrous remnants of extra-hepatic bile ducts and gallbladder with lymphocyte infiltration of the tissue. In the following weeks, biliary cirrhosis developed, accompanied by cystic bile retention, recurrent bile duct infections and malabsorption. Six months after the Kasai procedure, our patient underwent LDLT: the diseased liver was removed and segments II and III from our patient’s mother were used [4].

**Figure 1:**
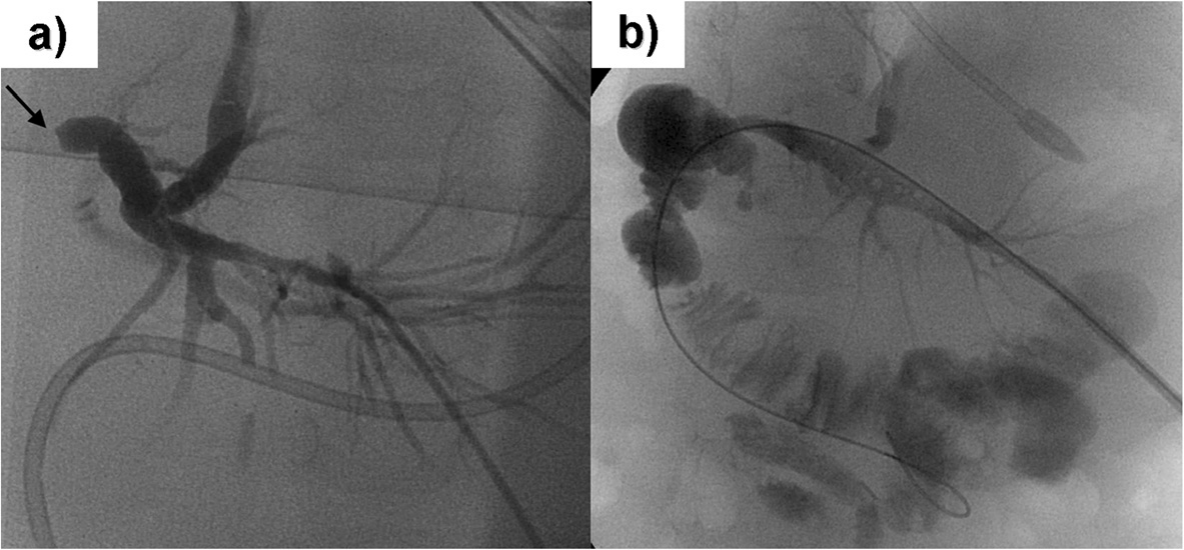
**Our patient, a 12-month-old boy with intra-hepatic cholestasis from occluded hepatico-jejunostomy after living donor liver transplantation. (a)** Fluoroscopy indicating trans-hepatic puncture of the segmental bile duct in liver segment III. The arrow indicates complete occlusion of the hepatico-jejunostomy. **(b)** Fluoroscopy indicating normal passage of the contrast medium to the jejunum after successful recanalization and balloon dilatation of the hepatico-jejunostomy.

Several revisions were necessary during the recovery period. Our patient presented with hypoplasia of the portal vein and inadequate flow from the portal anastomosis. Therefore, a porto-caval hemi-transposition was performed. The surgeon anastomosed an arterial inter-position graft from an ABO-identical deceased donor end-to-side to the recipient retro-hepatic vena cava and end-to-end to the portal vein of the liver graft. In order to increase portal flow, the inferior vena cava was ligated below the level of the transected liver veins of the recipient. Improvement of his overall condition, however, was not lasting. Over the course of two months following surgery, our patient developed occlusion of the hepatico-jejunostomy reflected by cholestasis and confirmed by ultrasound and X-ray (Figure 1a) imaging studies.

Our surgeon advised percutaneous trans-hepatic cholangiography and drainage (PTCD) under sonographic guidance, and was present when the procedure was carried out by our interventional radiologist. During PTCD a thin needle is inserted percutaneously and advanced through liver tissue under radiographic or sonographic guidance until a bile duct is entered. Non-ionic iodinated contrast is then used to outline the bile duct system. When the correct position of the needle is confirmed by X-ray, a catheter is inserted to allow the bile to drain either into a small pouch attached outside the body or into the small intestine.

Cholestasis subsided after an external bile duct drain (6 to 8F catheter; BARD^®^, Karlsruhe, Germany) was inserted and our patient’s general condition improved. However, persistent loss of bile via the drain increased his malabsorption, nutritional deficiency and weight loss. Further radiological interventions became necessary. Between the 12th and 17th months, five attempts were made to canalize the completely occluded hepatico-jejunostomy, since our surgeon did not deem it necessary to perform revision surgery unless unavoidable. After multiple attempts in 12 interventions under general anesthesia, catheterization through the hepatico-jejunostomy was finally successful.

Our radiologist dilated the stricture by balloon twice to a diameter of 5mm (Figure 1b). This effectively re-established the bile drainage to the gut. The percutaneous biliary drain could then be removed during the same intervention. In addition, the trans-hepatic access was embolized with coils to counteract the development of a peri-hepatic bilioma.

All PTCD interventions were accomplished under general anesthesia with mechanical ventilation. Anesthesia in a weak and malnourished child demands effective co-operation between anesthesia teams and pediatric intensive care unit (PICU) personnel during the peri-operative care period. Anesthesia was induced with short-acting drugs such as propofol and remifentanil and maintained with sevoflurane to keep potentially drug-induced central or hepatic toxicity low. Intervals of arterial hypotension, hypercarbia, hyperoxia, and hypoxia were meticulously averted. After each intervention our patient was transferred to the PICU where sedoanalgesia was tapered and our patient weaned from ventilation. His post-operative recovery periods were uneventful. No clinical signs of impaired neurodevelopment were observed.

Six months after the last PTCD our patient was well and the transplanted liver had gained full function as expressed by normal liver parameters (aspartate transaminase 56U/L, alanine aminotransferase 35U/L, γ-glutamyl transpeptidase, 59U/L, lactate dehydrogenase 296U/L, total bilirubin 0.37mg/dL). At two-year follow-up no further surgical and radiological interventions were found to be necessary.

## Discussion

Biliary leaks and strictures are common complications in pediatric patients with biliary atresia who have undergone liver transplantation [5]. Bile leaks or strictures at the anastomotic site or cut edge of the transected liver have been reported in 15 percent to 60 percent of recipients [4]. Non-surgical management of biliary complications is the preferred diagnostic and therapeutic modality, as operative repair is associated with significant post-operative morbidity [6,7]. Minimally invasive procedures such as balloon dilation and percutaneous drainage placement are safe and effective, but may require several attempts. Repeated re-interventions, however, increase the complication rate [8]. When PTCD fails, however, the follow-up protocol usually includes surgical revision of the transplanted liver.

Our patient was exposed to anesthesia 12 times during minimally invasive procedures. Anesthesia in a weak and malnourished child is quite a challenge to therapists. Effective co-operation between anesthesia teams and PICU personnel is crucial during the peri-operative care period. The child’s nutritional status has an impact on the peri-transplant outcome. Concurrent cholestatic liver disease contributes to fat malabsorption, with deficiency of calories and fat-soluble vitamins necessitating caloric assessment and supplemental feedings by tube or infusion [9]. Furthermore, immature immune systems in pediatric patients combined with immunosuppression increases the risk for infections and complications.

There is evidence of long-lasting cognitive impairment in babies from non-human primates exposed to general anesthesia. Administration of anaesthetic drugs during active synaptogenesis is associated with disorganization of cytoskeleton and impairment of dendritic branching. Developing glia cells are particularly sensitive to anesthesia-induced toxicity, manifested as stunted growth, delayed maturation, and disturbed process formation [10].

Results from animal studies suggest that apoptotic neuronal degeneration and late cognitive impairment is a potential long-term risk of early exposure to anaesthetic agents, but data from human studies are lacking. In particular, *N*-methyl-d-aspartate receptor antagonists and drugs that potentiate γ-aminobutyric acid signal transduction are potentially neurotoxic to the developing brain, especially when jointly administered [11]. Various domains of recovery such as physiologic, nociceptive, emotive, activities of daily living, cognitive, and overall patient perspective have been evaluated in adult-age surgical patients. Many patients had incomplete recovery by the third post-operative day [12]. Although not statistically significant, post-operative behavioral disturbance is more frequently observed in children less than two years of age [13]. The impact of general anesthesia, surgery and hospitalization upon cognitive, emotional and socio-behavioral development in children has not been well investigated. Most studies deal with pre-operative anxiety and predictors of increased anxiety [14].

In our patient’s case, anesthesia was induced with short-acting propofol and maintained with sevoflurane or propofol to avoid overt potential drug-induced hepatic impairment and neurotoxic side effects [15]. Post-operative recovery periods were uneventful. No clinical signs of impaired neurodevelopment were observed. Co-operation with parents and complication management by the multidisciplinary team of a surgeon, radiologist, pediatrician and anesthesiologist are necessary for successful treatment and care of children who develop intra-hepatic cholestasis following liver transplantation.

## Conclusions

Cholestasis arising from biliary stricture is a frequent complication in pediatric patients after liver transplantation. Minimally invasive procedures such as percutaneous drainage placement and balloon dilation are safe and effective, but may require several attempts before being successful. General anesthesia induced with short-acting propofol and maintained with sevoflurane or propofol is safe, as shown by the fact that no clinical signs of impaired neurodevelopment were observed.

## Consent

Written informed consent was obtained from the patient’s next-of-kin for publication of this case report and any accompanying images. A copy of the written consent is available for review by the Editor-in-Chief of this journal.

## Competing interests

The authors declare that they have no competing interests.

## Authors’ contributions

All authors were involved in the care of our patient. GP, WL and FJW performed the literature search and were the major contributors to the writing of the manuscript. PP, APC and WM revised the manuscript for important intellectual content. All authors read and approved the final manuscript.
